# Microbiome ecology and antibiotic treatment failure: resistance spread, microbial competition and community engineering

**DOI:** 10.1099/mgen.0.001856

**Published:** 2026-10-07

**Authors:** Myles Wheeler, Erik Bakkeren

**Affiliations:** 1 Department of Biological Sciences, University of Calgary, Calgary, Alberta, Canada; 2 Calvin, Phoebe and Joan Snyder Institute for Chronic Diseases, Cumming School of Medicine, University of Calgary, Calgary, Alberta, Canada

**Keywords:** antimicrobial resistance, community engineering, horizontal gene transfer, microbiomes, microbial competition

## Abstract

The failure of antibiotic treatment is a growing concern, driven in large part by the rising incidence of antimicrobial resistance (AMR). Increasing evidence points to the importance of microbial competition in preventing the colonization of disease-causing pathogens and the spread of AMR. Crucially, the pathogens we target with antibiotics do not exist in isolation but instead compete and interact with the microbial communities that colonize the human body, microbiomes. Here, we therefore argue that the key to approaching the challenge of antibiotic treatment failure is understanding the strategies and mechanisms that microbes use to succeed in microbiomes. We discuss how microbial competition influences the colonization of pathogens and the spread of resistance via horizontal gene transfer. We then discuss how microbial competition may be used to synergize with antibiotic treatment or even replace it. Overall, we argue that studying microbial interactions offers a powerful approach to understanding and even mitigating antibiotic treatment failure.

**Impact Statement**

A variety of factors contribute to antibiotic treatment failure. While genes found in pathogens that confer resistance to the antibiotics used are often cited as primary causes of antibiotic treatment failure, it is not simply the presence or absence of these genes that determines the outcome. This is because pathogens interact with both the host and the microbes with which they compete. In fact, recent evidence suggests that the outcome of antibiotic treatment is often different when microbes are targeted in isolation compared to within communities. Our review highlights the importance of microbiome ecology: the study of interactions that microbes have with each other and their host. We compile evidence from human microbiome, animal, and *in vitro* studies that support the importance of microbiome ecology in containing the spread of antimicrobial resistance and even replacing antibiotics. Microbiome ecology provides a framework for understanding why antibiotic treatment fails and for developing therapies that work with, rather than against, resident microbial communities.

## Data Summary

The authors confirm that all supporting data have been provided within the article.

## Introduction

 One of the biggest threats to modern medicine is the failure of antibiotic treatment and antimicrobial resistance (AMR) is the primary driver of this threat. AMR refers to genetically encoded determinants that render microbes that were once easy to kill with antimicrobials resistant. Indeed, AMR represents an escalating global crisis, currently contributing to the loss of 4.95 million human lives each year, with expectations that this burden will continue to rise to 10 million deaths per year by 2050 [1].

AMR is a well-recognized threat in disease-causing pathogens due to its ability to compromise the efficacy of antibiotic treatment. Yet, AMR can also be life-threatening if found in the microbes that normally colonize the human body, the human microbiome. Microbes can engage in horizontal gene transfer, leading to genetic information transfer from resistant cells to susceptible cells, conferring resistance and rapidly shifting our ability to control an infection with antibiotics [2]. Given the density of some human microbiomes, such as in our intestinal tract, they can serve as critical reservoirs of AMR. Indeed, the gut microbiome contains trillions of microbes made up of hundreds to thousands of species that all interact [3, 4] and, therefore, have the potential to assort AMR genes between species [5].

However, the ability of antibiotics to kill is not only determined by resistance genes but also by ecological context. Both microbe–microbe [6, 7] and host–microbe interactions [8, 9] can influence whether and how effectively antimicrobials kill and, therefore, contribute to antibiotic treatment failure. Even in the absence of resistance genes, these ecological interactions can determine the success or failure of antibiotic treatment. Regardless of the mechanisms underpinning antibiotic treatment failure, alternative strategies to manage infectious disease are becoming increasingly urgent. These strategies may stem from either alternatives to antibiotics or strategies to synergize antibiotic use with the natural ability of microbiomes to act as barriers to pathogens [10–13].

Microbial competition within microbiomes is abundantly common [14] and is central in determining the composition and function of a community [15]. We, therefore, argue that the key to the next generation of strategies to contain AMR is leveraging microbial competition. That is, to successfully design microbial therapeutics to synergize with or replace antibiotics, we must consider how the microbes we want to target naturally live: within microbiomes. We discuss how interactions in the microbiome influence the ability of pathogens and AMR strains to colonize the host, how microbiomes can modulate the spread of AMR, how microbiomes affect antimicrobial treatment, and how competition in the microbiome might be used to replace antibiotics to decolonize resistant strains. We focus here on the human intestinal microbiome, where species density and, in turn, interactions are most abundant, but we argue that the importance of microbial competition in minimizing antibiotic treatment failure will apply to other human microbiomes and discuss some examples throughout.

## Microbiomes provide a protective barrier against pathogens and AMR strains

Microbes colonize exposed surfaces throughout the human body, including the oral cavity, gastrointestinal tract, respiratory tract, urogenital tract and skin. These microbiomes act as ecosystems where microbes interact with each other and the host. Some microbiomes, like the vaginal microbiome, are low diversity, dominated by a few species of *Lactobacillus* [16], while others, like the gut microbiome [17], are incredibly dense, diverse and variable. A commonality, however, is that microbiomes across mucosal surfaces provide health benefits, including protection against the colonization of strains that can carry virulence (i.e. pathogens) or AMR [13, 16, 18–21]. This property is called colonization resistance. Here, we discuss colonization resistance as a general principle by which the microbiome can compete with both pathogens and AMR strains alike. In later sections, we discuss cases where the microbiome can specifically influence AMR itself. The mechanisms by which colonization resistance occurs are diverse (reviewed in detail in [22]) but can be broadly categorized as competition for resources or modulating the environment, including the host immune system, to make it more unfavourable for pathogens (Fig. 1).

**Fig. 1.**
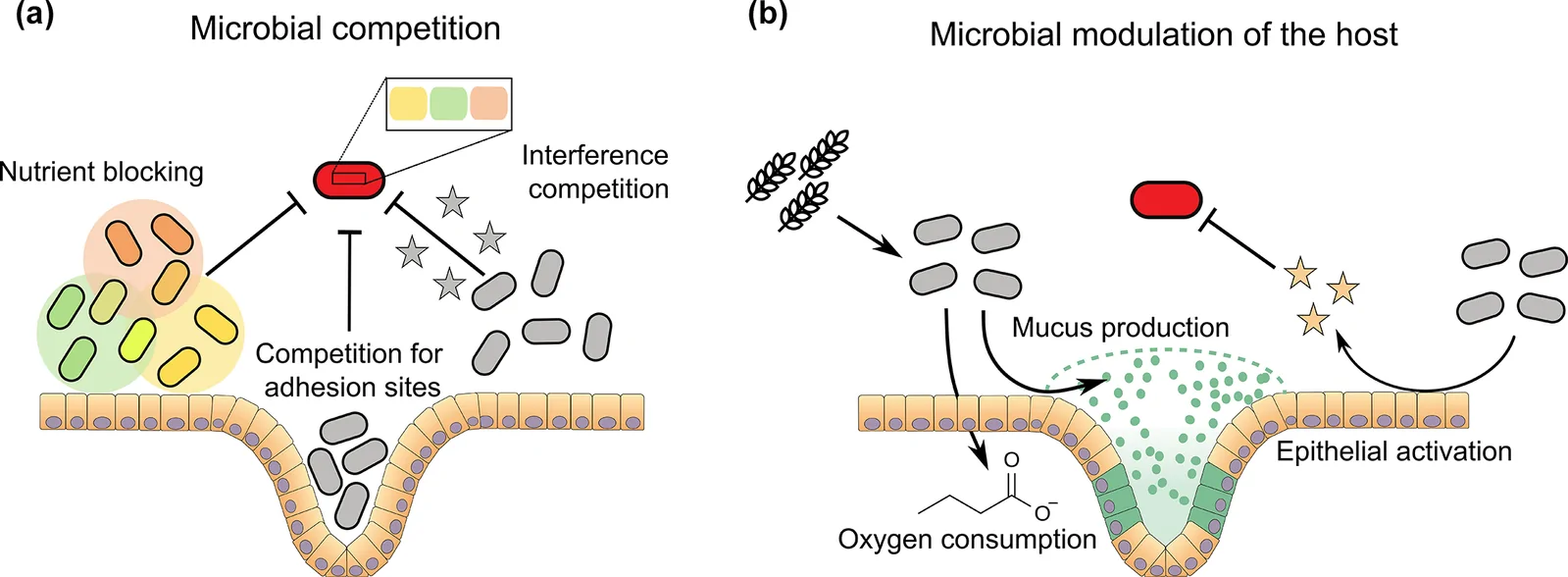
Microbiomes provide a protective barrier against pathogens, using the human intestinal microbes as an example. (**a**) Microbial competition. Resident microbes in the microbiome (depicted as grey, orange, yellow or green rods) restrict an invading strain (red rod) through two primary competitive mechanisms. The first is resource competition, where resident microbes occupy space, such as adhesion sites in the gut or where overlap in metabolic niches reduces the resource availability for invading strains. In the case of nutrient competition, the invader’s nutrient utilization profile (inset with coloured rectangles), determined by its genetic makeup, outlines its potential to grow on nutrients (depicted as coloured circles). Microbes in the microbiome that use these nutrients can block access of the invading strain to those nutrients (microbes are coloured to depict nutrient preference) in a phenomenon called nutrient blocking. The second is interference competition, including bacteriocins (grey stars) and contact-dependent systems that directly inhibit competitors. (**b**) Microbial modulation of the host. Microbes can metabolize dietary fibre (wheat icon) into short-chain fatty acids, such as butyrate (chemical structure shown). Butyrate drives beta-oxidation, consuming intestinal oxygen and thereby restricting the expansion of facultatively anaerobic pathogens. Microbes can stimulate goblet cells (green cells) to increase mucus production. Mucus (green dots) acts as a physical barrier preventing bacterial contact with the epithelium (beige cells) while serving as a scaffold enriched with antimicrobial peptides, antibodies and microbes that further reinforce colonization resistance. Microbes can also activate epithelial cells, leading to the release of antimicrobial peptides (beige stars) which can inhibit invading bacteria.

## Competition for resources is central to determining microbial invasion

In the gut, the composition of a healthy microbiome is highly variable at the species level [17, 23, 24] and is even more variable at the strain level [25]. What appears to be consensus is that diverse gut microbiomes are associated with health, but the compositions of microbiomes that confer these health benefits are not always the same [13, 24, 26–29]. Indeed, we still lack a complete understanding of what drives the success of a given strain or species in a community.

One strategy is to search for commonalities in microbial needs that promise to be much more conserved than species compositions (reviewed in [15]). For example, all microbes need resources to grow, such as nutrients and space [3]. Given that there are finite nutrients available for microbial consumption, microbes must, therefore, compete for nutrients via dedicated metabolic pathways or strategies to access spatially privileged sites, known as resource competition.

As two species that compete for the same limiting resource cannot coexist [30], it is expected that natural selection will promote diversification of metabolic repertoires so that microbes can establish a niche. The metabolic diversity that is found in species that inhabit the microbiome supports this intuition. For example, *Bacteroides* spp. that colonize the gut can sometimes carry hundreds of carbohydrate-active enzymes, and each species or strain can carry unique enzymes [31]. Similarly, the pangenome of *Escherichia coli* is large, and different strains of *E. coli* often use different metabolic genes [32–34].

The consequence of metabolic diversification on the invasion of new strains is, therefore, that competition for nutrients in microbiomes is a collective effect: an invading strain can only reliably establish if the community as a whole does not consume all the available nutrients that the invading strain needs, a process called ‘nutrient blocking’ [13] (Fig. 1a). And, the metabolic genes at disposal for the invading strain relative to the community must correlate with the available nutrients at a given time. [15, 35]. Indeed, changes in the available nutrients have been shown to influence the ability of pathogens to colonize microbiomes, such as the effect of diet shifts on the gut microbiome [36], but also in other microbiomes, such as on the skin [37] or in the vagina [38]. For example, during menstruation glycogen levels are reduced, leading to a reduction in *Lactobacillus spp.* that feed on glycogen and normally provide resistance to colonization against pathogens [38].

In addition to nutrients, spatial structure influences competition in the microbiome. In the intestinal tract, there are anatomical structure changes from the stomach to the small intestine to the colon, but there are also micron-scale structures throughout [39]. For example, invaginations in the intestinal wall can separate species that otherwise would compete, or the epithelium and fibre particles can support species that have evolved mechanisms for adherence or motility, such as pili or flagella [40], while excluding others. In the latter case, microbes that have similar adaptations would compete for adhesion sites and space (Fig. 1b). Generally, however, the effect of spatial structure is to reduce the strength of interactions, facilitating global co-existence. For example, a species that competes strongly with a pathogen for access to a specific nutrient may provide no colonization resistance at all if the pathogen can access spatially privileged sites next to the epithelium or along the mucus layer, as shown for the gut pathogen *Salmonella enterica* serovar Typhimurium (*S*. *Typhimurium*) [41]. However, the overall relative contribution of spatial structure to colonization resistance mediated by the microbiome is not well understood, nor whether the carriage of AMR genes influences competitiveness for access to spatially privileged sites.

## Interference competition can provide a competitive edge for resources

Blocking of spatial and nutritional resources is not the only direct mechanism by which the microbiome can compete with invading pathogens, however. Bacteria have evolved an arsenal of competition systems or ‘weapons’ to kill other microbes [42]. This form of competition is called interference competition and is distinct from resource competition (Fig. 1a). The mechanisms by which interference competition work in bacteria are diverse and include contact-dependent weapons, such as Type 6 Secretion Systems (T6SS) or diffusible toxins, such as bacteriocins [11, 42, 43]. Because these weapons are costly to produce [42, 44], they are expected to target niche competitors and thus counter the energetic burden of production. Indeed, the spectrum of activity of many of these toxins is narrow [45], and metabolic similarity is a reasonable proxy for the likelihood of being killed by interference competition [46, 47]. However, some mechanisms of interference competition are broad in spectrum [45].

Given the potency and diversity of these bacterial weapons, it is not surprising that there is evidence that these mechanisms contribute to colonization resistance. For example, resident *Bacteroides fragilis* use T6SS to restrict colonization by enterotoxigenic *B. fragilis*, a common pathogen associated with intestinal colitis [48, 49]. Likewise, the lantibiotic produced by *Blautia producta* can protect against colonization of vancomycin-resistant *Enterococcus faecium* [50]. These examples illustrate how both contact-dependent and diffusible weapons can enhance microbiome-mediated protection against pathogens.

Beyond direct microbial competition, which has evolved to provide a competitive edge for competing species [46], microbes within microbiomes can produce inhibitory products as a byproduct of metabolic processes that can inhibit invading strains. An example in the intestinal tract is short-chain fatty acids (SCFAs), which are byproducts of microbial fermentation by obligate anaerobes. SCFAs are also known to contribute to colonization resistance: propionate produced by *Bacteroides spp.* can inhibit *S*. *Typhimurium*, a common AMR-associated strain [51]. This metabolism-mediated inhibition also occurs in other microbiomes, such as lactic acid production by vaginal *Lactobacillus* spp*.* [52]. Although, these metabolites arise as by-products of metabolism rather than evolved antagonistic systems, they nevertheless provide resident microbes with a competitive advantage to exclude pathogens and are expected to limit the establishment of AMR-carrying strains too.

## From colonization resistance mechanisms to limiting AMR strain invasion

Mechanisms of colonization resistance have been characterized using mainly susceptible pathogens and have often not explicitly tested how carriage of resistance genes affects outcomes. However, we argue that how a microbiome protects against invasion of a new strain is general and principles are expected to govern pathogens, AMR strains and beneficial microbes alike. This suggests that understanding the rank importance of different mechanisms of colonization resistance and their interaction can provide the foundation for understanding how microbiome ecology influences AMR persistence, colonization and spread.

We must also identify the contexts in which each form of competition is most successful. For example, microbiomes can also indirectly restrict pathogen colonization by modifying the host environment (Fig. 1b). Species within the gut microbiome maintain intestinal anaerobiosis via the production of SCFAs, which act as a signal for intestinal cells to switch their metabolism to beta-oxidation. This process consumes oxygen and in turn prevents blooming of facultatively anaerobic pathogens [53]. Similarly, the microbiome also reinforces epithelial barrier function, stimulates mucus production and promotes the production of antimicrobial peptides, collectively creating a host environment that is less permissive to colonization by invading microbes [54–56]. This means that microbial competition will work in tandem with host effects, and their effects are sometimes difficult to disentangle.

Even among direct forms of microbial competition, their relative importance, and their interaction is unclear. For example, while the roles of resource and interference competition on colonization resistance have been studied separately, their interaction is less clear. It is likely that both forms of competition synergize to optimally protect the microbiome from invasion by a range of pathogens, and perhaps each form can act to replace the other in times of perturbations by dietary changes, inflammation or antibiotic treatment. Indeed, a series of studies seem to suggest that the ability of *Clostridium scindens* to protect against *Clostridioides difficile* is mediated by nutrient competition in some contexts and via bile acid secondary metabolism in others [57–60]. Instead of synergizing, however, interference competition and nutrient competition may also act antagonistically, providing windows of opportunity for pathogens or AMR-carrying strains colonizing the microbiome. For example, the production of broad-spectrum bacteriocins can kill members of the microbiome, alleviating nutrient blocking, mirroring circumstances where microbiomes are treated with a variety of broad-spectrum antibiotics and drugs [61, 62]. A recent study demonstrated that *Blautia pseudococcoides* producing a lantibiotic hinders the recovery of the gut microbiome following antibiotic treatment, reducing colonization resistance to *Klebsiella pneumoniae* and *C. difficile*, [63] and it is expected that these effects will translate to AMR strains too. This is because a pathogen can rapidly shift from an antibiotic-susceptible pathogen to an AMR pathogen (discussed in the next section).

In addition, because resistance mechanisms in AMR strains often impose fitness costs [64, 65], the mechanisms that drive colonization resistance towards pathogens may be even more potent against pathogenic AMR strains.

## Microbiomes can be reservoirs of AMR and promote its spread

While microbiomes provide a barrier for invading microbes, a microbiome’s composition can shift following a perturbation and allow the invasion of an AMR strain [66, 67]. Because many resistance genes are carried by non-pathogenic strains, their presence can go unnoticed. Indeed, microbiomes can harbour many microbes containing AMR genes without causing disease. Although AMR genes impose fitness costs due to the energetic burden of their maintenance and expression, they can nevertheless persist in microbiomes through a combination of antibiotic selection and horizontal gene transfer. We first discuss the dynamics of AMR spread in the absence of antibiotic selection and then discuss how perturbations, including via antibiotics, affect AMR spread.

AMR genes are often found on mobile genetic elements, such as plasmids, integrative conjugative elements or even bacteriophages (bacterial viruses; phages) [2]. This means that there is potential for horizontal gene transfer, which can rapidly assort genetic information between species and promote persistence of AMR genes within microbiomes. For example, transfer of plasmids can be sufficient to maintain AMR genes even in the absence of antibiotic selection [68]. It also means that AMR can be transferred to pathogens which can then lead to antibiotic treatment failure.

Plasmids are particularly potent drivers of AMR because they can carry multiple resistance genes and disseminate them between bacterial strains and, in some cases, across diverse bacterial species via the contact-dependent transfer of DNA, conjugation [69]. For example, the plasmid encoding New Delhi metallo-β-lactamase-1 (NDM-1) carries multiple AMR genes and has been identified across diverse gut-resident Enterobacteriaceae species, including *K. pneumoniae*, *Enterobacter cloacae*, *Proteus spp.*, *Citrobacter freundii* and *Klebsiella oxytoca* [70]. Furthermore, human and mouse studies have demonstrated that multidrug AMR plasmids, such as NDM-1- or extended-spectrum beta-lactamase (ESBL)-encoding plasmids, can conjugate between strains and species [71], even in the absence of antibiotic selection [72].

Generally, high-density environments significantly increase the likelihood of interactions between cells (Fig. 2a). By extension, the intestinal tract is thought to be a primary reservoir for AMR, driven by its capacity to facilitate interactions between species due to its density and diversity [5]. And, ecology is thought to be a principal driver of horizontal gene transfer in microbiomes, with species that co-inhabit the same site in the human body more likely to exchange genetic information than those at disparate body sites or are adapted to different niches within the same body site [73].

**Fig. 2.**
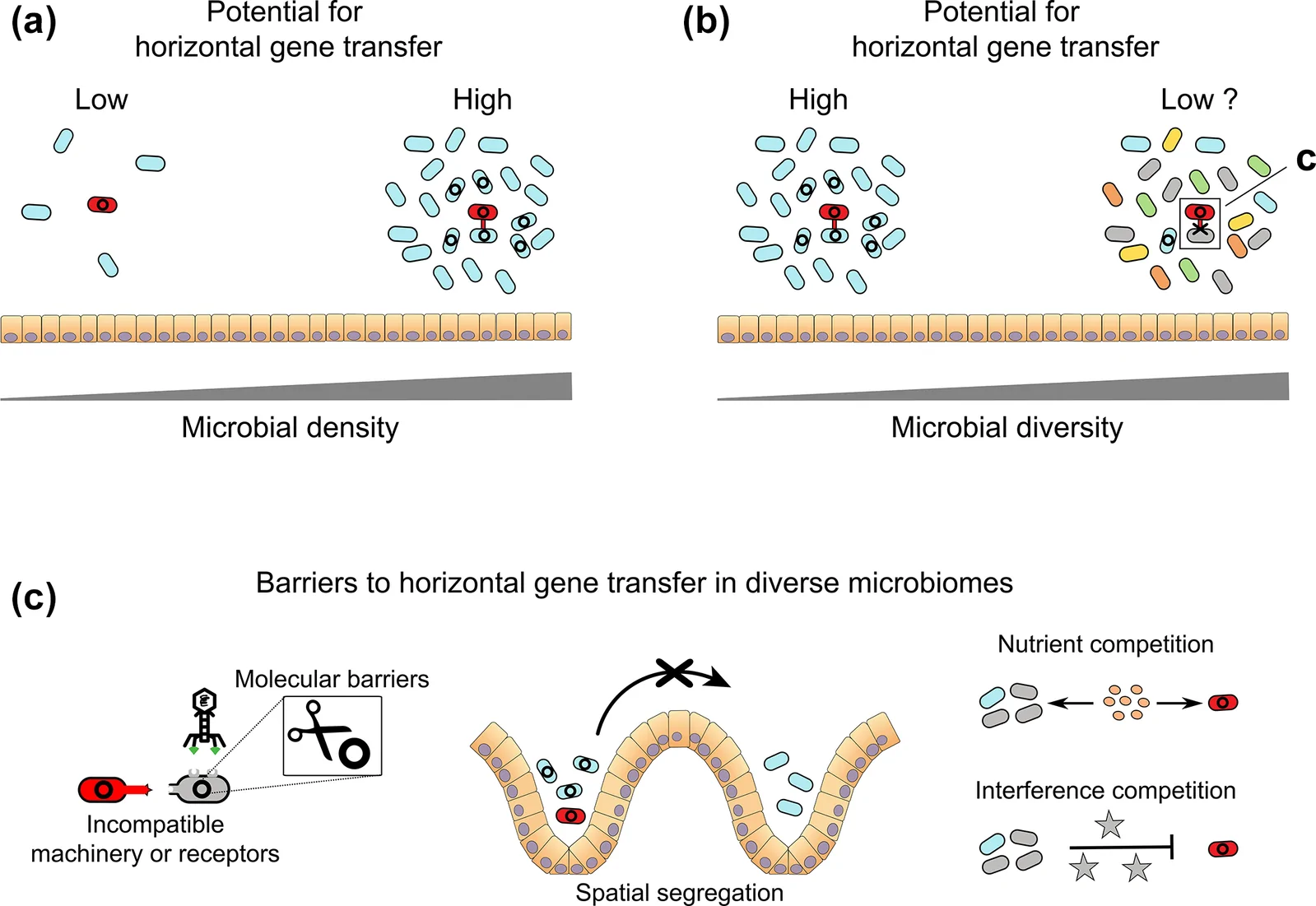
Microbiome ecology influences the spread of AMR via horizontal gene transfer. (**a**) Increasing microbial density elevates the probability of horizontal gene transfer by increasing the likelihood of cell-to-cell contact to occur between plasmid donor (red rods) and recipients (blue rods). The example of a plasmid (black circles) spreading from a donor to a recipient to form a transconjugant (recipient with a plasmid) is shown. (**b**) Increasing microbial diversity (differently coloured rods represent different species) may reduce the potential for horizontal gene transfer. While dense communities may still permit donor-recipient interactions, diverse communities introduce ecological constraints limiting plasmid spread. The inset (**c**) highlights these constraints. (**c**) Diverse microbiomes impose multiple constraints on horizontal gene transfer. Horizontal gene transfer can be limited between interacting species via molecular barriers, including defence systems (scissors; e.g. CRISPR-Cas and restriction-modification systems) that recognize and degrade incoming plasmid DNA. Horizontal gene transfer may also fail due to incompatible conjugation machinery in the case of plasmids or the lack of surface receptors in the case of phage transfer. Ecology also imposes barriers to horizontal gene transfer. Spatial segregation limits physical contact between compatible partners. Furthermore, microbial competition, whether via nutrient competition (nutrients represented as orange dots) or by interference competition (grey stars) may limit densities of compatible partners for horizontal gene transfer.

## Molecular barriers to horizontal gene transfer in microbiomes

However, beyond the necessity for co-localization for gene exchange, mobile genetic elements do not transfer with equal rates to all species (Fig. 2b–c). Phages require dedicated cell surface receptors, which may partially explain why the contribution of phage transfer to AMR spread is estimated to be lower than plasmid spread [74]. But plasmids, too, often have a principally narrow host range. For example, conjugation machinery between Gram-negative and Gram-positive microbes is distinct as they have different cell wall structures [69, 75]. Furthermore, plasmid replication origins and replication proteins have evolved to work with specific host replication machinery, resulting in conjugation most commonly occurring between closely related species [69]. There are, however, also cases where plasmids can be transferred between distantly related species, such as between Gram-negative and Gram-positive microbes in microbiomes [76]. These cases appear to be the minority, with most plasmids having a bias for transfer to phylogenetically similar species [76].

In addition to differences in mechanisms of conjugation of distantly related microbes, there are molecular barriers that bacteria have evolved to restrict the acquisition of foreign genetic information. This is because, while horizontal gene transfer can provide fitness benefits in some contexts, it can impart metabolic costs to the cells that receive them [64], or, in the case of phages, lead to cell death as the phage replicates. These molecular barriers are discussed elsewhere [77, 78], but can either act to target frequently occurring patterns on foreign genetic material like unmethylated DNA in the case of restriction modification systems or can target specific sequences, such as CRISPR-Cas systems, analogous to the adaptive immune system in humans.

## Competition in the microbiome as a barrier to horizontal gene transfer

Collectively, these molecular barriers mean that the rate of spread of mobile genetic elements is affected by ecological processes within microbiomes (Fig. 2b–c). The composition of a microbiome affects both the densities and gene regulation of interacting species, altering the likelihood of transfer. As a result, the spread and persistence of AMR plasmids are largely shaped by community structure, host diversity, spatial organization and interspecies interactions [79, 80]. In this subsection, we discuss the effect of competition in the microbiome on AMR spread in the absence of antibiotic selective pressure.

In the case of conjugation, cell-to-cell contact between a donor and recipient is needed. This means that, in the absence of antibiotic selective pressure, the same ecological processes that affect invasion of new strains, such as the universal need for microbes to acquire resources, will likely restrict the spread of AMR. This expectation is because a newly invading plasmid-carrying strain must establish to sufficient density to encounter a recipient strain. Once transferred into a well-established resident strain, however, AMR plasmids may continue to disseminate even if the invading donor remains rare [81], although this will depend on the density of the recipient [82]. Similarly, a new potential recipient can only receive a plasmid if it can invade a microbiome to sufficient densities. Illustrating this point, two strains of *S. Typhimurium* could only engage in efficient plasmid transfer in the intestine of mice if they could use slightly different carbon sources, which facilitated their co-existence and, in turn, plasmid transfer [83]. This suggests that competition for nutrients can be an important driver of plasmid transfer dynamics, similarly to its importance in colonization resistance [13].

The host can also provide a structured environment as seen in pores on the skin [84] or the mucus layers and crypts found in the intestinal tract [39]. Because microbial populations can then be segregated into distinct spatial niches, microbes are forced to interact primarily with their immediate neighbours rather than with the entire microbiome. As a result, while local patches may engage in repeated plasmid exchange between compatible partners, spatial segregation can limit global spread of resistance genes throughout the microbiome [85].

In addition to resource competition, interference competition has the potential to modulate the densities of microbes that can engage in horizontal gene transfer. For example, microbes that produce bacterial weapons can kill plasmid-carrying strains, reducing the potential for conjugation. Similarly, potential recipients can also be killed before they are able to acquire a mobile genetic element [86]. Contrarily, interference competition can also increase the rates of horizontal gene transfer [87]. In *Vibrio cholerae*, the T6SS can lyse target cells, causing them to release DNA, which *V. cholerae* can then uptake [87]. In that case, both the T6SS and genes important for uptake of DNA are regulated in response to chitin, an abundant biopolymer found in crustaceans, algae and fungi, suggesting its role in aquatic environments. While *V. cholerae* is also a human gut pathogen [87], its role in interference competition in facilitating DNA transfer in the gut is so far unclear, yet precedence for this role in other systems reveals intriguing potential. Lastly, the effect of interference competition on horizontal gene transfer is likely to depend on the spectrum of activity of the bacterial weapon, and the susceptibility of the strains engaging in horizontal gene transfer. For example, while weapons that eliminate plasmid-carrying strains or recipients can reduce conjugation, broad-spectrum antibiotic toxins, such as those produced by *B. pseudococcoides* can disrupt gut microbiome diversity [63]. In that case, broad-spectrum weapons may weaken colonization resistance and in turn promote rapid expansion of interacting strains and in turn transmission, particularly if strains are resistant to the weapon used.

Regardless of the mechanism, microbial diversity imposes competition on microbes engaging in horizontal gene transfer, which can restrict the probability of transfer (Fig. 2b–c). Therefore, while the density and diversity of some microbiomes like the human gut microbiome can provide a hotspot for horizontal gene transfer and assortment of genetic information to diverse microbes [5], microbiome diversity itself may act as a tempering force via ecological competition. This can occur via resource competition, interference competition or indirect mechanisms via the host, reducing the population sizes of specific species where the rates of horizontal gene transfer are the greatest. However, more work is needed to explore the mechanisms and ecological contexts by which microbial diversity can both facilitate horizontal gene transfer and restrict it and how this varies depending on the specific resistance genes.

## Microbiome perturbations can alleviate barriers to horizontal gene transfer

Perturbations to the microbiome can shift the composition of a microbiome, opening a window of opportunity for invading microbes, including those that contain resistance genes. The most direct example is the perturbation of the human gut microbiome via antibiotics, where selection for resistance genes is generated by the antibiotic, increasing plasmid spread [88]. But antibiotics can boost plasmid spread even if the AMR genes on the plasmid do not confer resistance to that antibiotic [72]. This occurs via a compositional shift that can lead to overgrowth of certain strains, and if they carry them, resistance genes that can then be transferred [89]. Indeed, in intensive care unit patients that are frequently given antibiotics, blooms of *Enterobacteriaceae* and high rates of AMR can be observed [90].

But the effect is expected to be dependent on the spectrum of activity of the antibiotic. For example, broad-spectrum antibiotics, such as late-generation fluoroquinolones or cephalosporins disrupt diverse members of the microbiome [91] and are expected to lead to vast compositional shifts. In contrast, narrow-spectrum antibiotics, such as fidaxomicin that can target *C. difficile* with reduced collateral damage to the microbiome compared to broad-spectrum antibiotics [92], will likely preserve the barriers to AMR strain colonization and horizontal gene transfer.

In addition to antibiotics, perturbations, such as dietary shifts and inflammatory responses can have similar effects. For example, a ‘Western diet’ that contains a low amount of fibre and a high proportion of simple sugars can cause the expansion of species, such as *E. coli* that thrive in these conditions, and loss of species that rely on dietary fibre [93]. Furthermore, the fat in a Western diet causes the host to release bile acids into the intestine, which can inhibit many strict anaerobic species while being tolerated by *Enterobacteriaceae*, fuelling blooms and plasmid exchange [66]. These diets can also trigger inflammation, which generates electron acceptors, such as nitrate, tetrathionate and reactive oxygen species in the gut, allowing facultative anaerobes, such as *E. coli* and *S*. *Typhimurium* to respire and bloom [94, 95]. Indeed, inflammation caused by *S*. *Typhimurium* can directly boost conjugation between *Enterobacteriaceae* via inflammation-mediated suppression of microbiota diversity and corresponding blooms of *Enterobacteriaceae* [96].

Perturbations can also affect the regulation of horizontal gene transfer. For example, in *Enterococcus faecalis* that colonizes the intestinal tract, plasmid-free recipients secrete a recipient pheromone peptide, cCF10, activating the expression of conjugation genes in a donor strain [97]. This way, plasmid transfer only proceeds in the presence of sufficient recipients. As another example, indole, which is a byproduct of tryptophan metabolism produced by gut species, such as *E. coli* and *Bacteroides thetaiotaomicron*, can inhibit conjugation in *E. coli* [98]. Finally, colibactin, which is produced by some strains of *E. coli,* can initiate DNA damage in other cells, inducing stress responses [99, 100]. Stress responses, such as the bacterial SOS DNA-damage response, are known to induce prophages [99]. Additionally, stress responses can control the regulation of conjugation machinery for integrative conjugative elements that frequently encode AMR genes. Integrative conjugative elements are chromosomally integrated but can excise and engage in horizontal gene transfer via conjugation when induced by the SOS response [101]. In all these examples, perturbations that shift the distribution of pheromone-, indole- or colibactin-producing microbes would alter the rates of horizontal gene transfer in microbiomes.

## Leveraging microbiome ecology to target pathogens and reverse AMR

The spread of AMR has the potential to make antibiotic treatment difficult and, in some cases, impossible. However, changes in the relative contribution of the ecological drivers of AMR spread, including shifts in nutrient availability, microbial competition and the host can be sufficient for strain turnover in human microbiomes. Strains of the same species, for example *E. coli* in the human gut [102] or *Staphylococcus epidermidis* in the skin [103], can replace each other over time. Evidence of this pattern can be seen in travel-return studies. In a study following nearly 2,000 participants during their travels, ~34% were colonized by ESBL-encoding *E. coli* after travelling abroad from Europe, and only 11% retained this AMR *E. coli* 12 months after returning home without a dedicated intervention to remove the AMR *E. coli* [104]. Collectively, these findings indicate that although the gut microbiome is generally stable throughout adulthood, we can find evidence of turnover. This suggests that changes in the microbiome can be sufficient to exclude strains that carry AMR.

Another line of evidence that the microbiome has the potential to compete with strains is via faecal microbial transplants. Reintroduction of microbiome diversity through a faecal microbial transplant from healthy donors leads to 85% success in preventing recurrence of infections caused by *C. difficile*, surpassing the success of antibiotic therapy [105]. Furthermore, a meta-study on the decolonization success of AMR pathogens, such as *K. pneumoniae, E. coli* or *E. faecium* from the human gut reported success rates around 48% [106]. But, while faecal microbial transplants suggest that competition in the microbiome can be leveraged to decolonize AMR strains by introducing microbes that establish and competitively displace resistant strains, reproducibility is challenging. Indeed, faecal microbial transplants represent a broad and poorly defined intervention, with microbial, metabolic and phage components transferred in tandem [107]. In addition, the microbiome in the patient receiving the faecal microbial transplant will provide colonization resistance towards the microbes in the transplant, affecting which strains establish and which ecological interactions drive displacement [108]. Defined consortia, molecules or targeted interventions, however, have the potential for more controlled and predictable effects, and recent studies [34, 109–112] detailed below, have begun to identify the ecological mechanisms underlying microbiome-mediated decolonization.

## Competition for nutrients can drive competitive exclusion

In general, the mechanisms that block invasion of newly arriving microbes into microbiomes and the mechanisms that promote decolonization of a pre-existing microbe are expected to overlap. This is because in both cases, competition with other microbes is central to the fitness of a strain. However, a key difference is relative abundance. When a strain is invading a microbiome, it is at a density disadvantage compared to the rest of the microbiome, referred to as a priority effect [113]. Nutrient blocking, for example, is expected to be especially relevant for protecting a microbiome against invasion of an AMR strain, as the microbiome can use available nutrients before the invading strain [13]. For decolonization, however, microbial consortia must compete for nutrients with a pre-established strain, no longer benefiting from a priority effect. This makes it much more challenging to leverage nutrient competition for decolonization, unless the microbial consortia can use nutrients more efficiently than the target strain or be introduced at high abundance repeatedly (Fig. 3a).

**Fig. 3.**
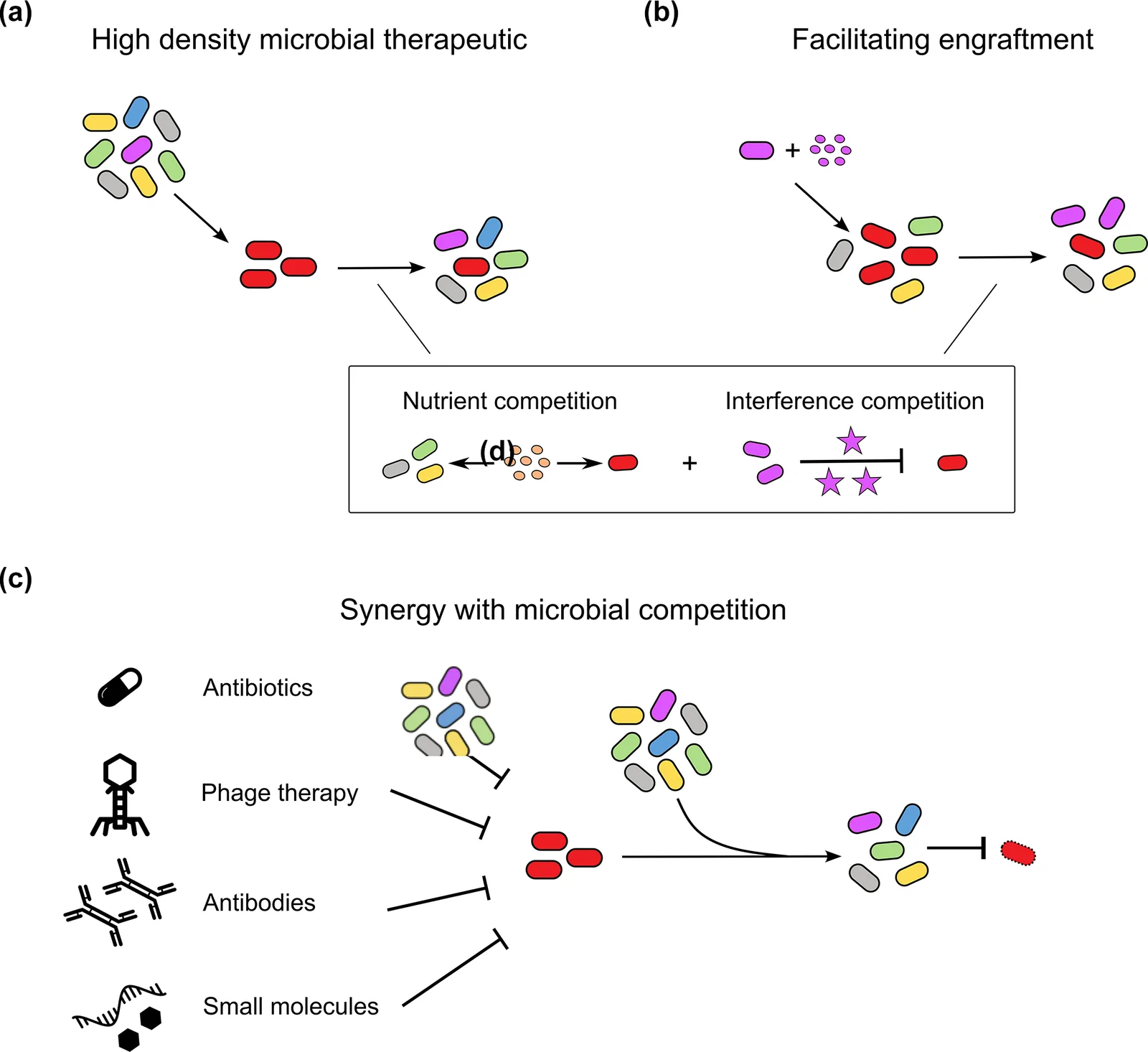
Leveraging microbial competition to target pathogens. (**a**) Administration of high-density microbial therapeutics introduces a community (depicted as multi-coloured rods) that suppresses pathogens (red cells) through a possible combination of competitive mechanisms including nutrient competition (orange dots) and interference competition (purple stars), as shown in the inset. (**b**) As an alternative to a high-density consortium of microbes, engraftment of a therapeutic microbe (purple rod) may be enhanced through co-administration of a private nutrient (purple dots) for the therapeutic microbe. In this case, a high density can be achieved via nutrient supplementation. Once established, the microbe may suppress a pathogen via a possible combination of competitive mechanisms, as in panel a. (**c**) Microbial competition can be combined with interventions to enhance clearance of invading strains. These include antibiotics, bacteriophage therapy, antibodies and small molecules, such as antisense oligonucleotides (single-stranded nucleic acid) or antimicrobial peptides (hexagons). These approaches reduce the pathogen’s abundance or fitness, and microbial competition, provided by either an administered microbial consortium or the resident microbiome, can finish off struggling pathogens.

Nevertheless, nutrient competition has been shown to be central in facilitating decolonization in the absence of antibiotics. For example, *E. coli* strains isolated from healthy humans could facilitate the decolonization of AMR *E. coli* in mice via competition for carbohydrates, but only in combination with a diverse microbiota [111]. Moreover, introducing *K. oxytoca*, which consumes a broader range of carbon sources compared to that of *E. coli*, expanded the range of AMR *E. coli* strains that could be decolonized. In a second study, a consortium of 18 gut microbiota species could facilitate decolonization of AMR *E. coli* and *K. pneumoniae* from the gut [110]. They identified gluconate, a carbon source, as a key driver of competitive exclusion. The 18-species consortium provides competition for gluconate with *K. pneumoniae*, causing *K. pneumoniae* to switch to less preferred carbon sources and ultimately facilitating its decolonization. Also in this case, efficient decolonization required all 18 species [110], indicating that this process is not driven by one species but rather by a collective effect. Lastly, nutrient competition mediated by the microbiome can also specifically outcompete AMR strains due to fitness costs arising from resistance mutations [114]. A recent preprint demonstrated that carbapenem-resistant *K. pneumoniae* carrying a mutation in a glycoporin are at a competitive disadvantage in the presence of a community that consumes glycerol-containing compounds [115].

Overall, these studies suggest that mechanisms similar to nutrient blocking can facilitate decolonization of species even if the therapeutic microbial strain or consortia facilitating decolonization does not have a priority effect. However, future work should investigate relative growth rates and how sensitive this process is to relative densities between competing strains to understand the mechanistic basis of this nutrient-based competitive exclusion.

## Bacterial warfare in microbiomes can mediate competitive exclusion

Like nutrient competition, interference competition is affected by cell density. Diffusible toxins, for example, are most effective at high cell density, where sufficient concentrations can accumulate to inhibit competitors [116]. This, therefore, suggests that ecology will determine if a given bacterial weapon will be effective: a strain defending its niche may be more likely to deploy a toxin than one that is invading its niche. In contrast to diffusible weapons, contact-dependent weapons have the potential to facilitate invasion as they can antagonize neighbouring cells one-at-a-time [117]. These predictions appear to be borne out in microbiomes, where pathogens, such as *S*. *Typhimurium* or *V. cholerae* use contact-dependent weapons like T6SSs to promote their invasion into the gut microbiome [118, 119]. Therefore, the ecology of bacterial warfare is important for understanding how to best leverage competition for our advantage. Perhaps the best strategy for targeted displacement of an existing AMR strain is to leverage a contact-dependent weapon.

However, interference competition can interact with other forms of competition to influence the outcome of bacterial warfare. Indeed, despite nutrient competition and interference competition largely being studied independently, these mechanisms frequently operate simultaneously within complex microbial communities [3, 15]. For example, as the benefits of diffusible toxins scale with density, if a strain can gain a foothold using other means, its toxin can provide a strong competitive advantage and displace a competitor. Theory and experiments converge to suggest a role of metabolism and nutrient availability in shaping the outcome of toxin-based competition: if a strain can colonize dependent on its metabolism, it can leverage a diffusible toxin to displace a competitor, whereas in the absence of key metabolic pathways to facilitate its growth, the toxin is dispensable [34] (Fig. 3b). In support, there is evidence for the utility of toxins when pathogens and symbionts co-bloom and both reach high densities. For example, during inflammation, *E. coli* co-bloom with *S*. *Typhimurium* and can compete using diffusible bacteriocins, such as microcin H47 or M and colicin Ib [120, 121].

This indicates that, therapeutically, a microbe that can antagonize a target AMR strain may be most effective if it is either equipped with a metabolic pathway that can allow it to colonize [34] or is introduced at high density, for example by a daily high-density microbial supplement [122] or purified antimicrobial toxins [123]. While the latter of these strategies are transient and require re-dosing, they have the potential advantage of engineering microbiomes transiently rather than introducing a microbe that has the potential to persist for long periods of time [109]. More work is needed to compare the effectiveness of high-density microbial therapeutics to a microbial therapeutic that engrafts into a microbiome (Fig. 3a, b). And, at present, both approaches are still less effective than antibiotics [124].

## Synergy between microbial competition and therapeutic interventions

Microbial competition can shape microbiomes, and we, therefore, have potential to leverage it to target AMR strains. However, pathogens and AMR strains will also have their own strategies to compete in microbiomes. Therefore, it is probable that the most successful strategies will tip the balance of competition in the microbiome’s favour. This can take the form of synergizing antibiotic treatment with microbial competition or leveraging the immune response, non-antibiotic drugs and small molecules or synthetic biology approaches (Fig. 3c).

## Antibiotic treatment

As antibiotics are often broad in spectrum, the gut microbiome can remain perturbed for long periods of time after treatment. Indeed, antibiotic treatment can take up to 1.5 months to recover to pre-treatment levels in terms of broad diversity [125], and signatures of prior antibiotic treatment can be detected up to 8 years later [109]. This can cause long-lasting collateral damage to the microbiota (reviewed in [89] and [126]), providing a window of opportunity for pathogen colonization or recurrence.

The ecological consequences of antibiotic treatment also depend on antibiotic class and spectrum of activity. Broad-spectrum antibiotics, such as late-generation fluoroquinolones or cephalosporins, can remove diverse members of the resident microbiota, disrupting beneficial interactions that may synergize with antibiotic treatment, such as resource or interference competition [126]. Narrow-spectrum antibiotics are, therefore, appealing as they have the potential to preserve the microbiome. Vancomycin and metronidazole are often termed narrow-spectrum due to their inability to kill most Gram-negative microbes, yet they still target a range of Gram-positive microbes and disrupt microbiome diversity [127].

As an example of the consequences of antibiotic collateral damage on the microbiome, *C. difficile* can be treated by antibiotics, but its ability to form spores allows it to form reservoirs of recalcitrant cells in the gut that can regrow after the withdrawal of antibiotics. This is because antibiotics decimate the microbiome and *C. difficile* can thrive in the post-antibiotic gut in the absence of competitors. However, reintroduction of microbiome diversity via a faecal microbial transplant from healthy donors or even defined bacterial consortia have been shown to prevent recurrence of *C. difficile* in patients [105, 128]. In line with this, antibiotics that target *C. difficile* with a narrow spectrum of activity and preserve the microbiome, like fidaxomicin, appear to reduce recurrence [92]. Similarly, gut pathogens like *S*. *Typhimurium* do not form spores but can instead form reservoirs of persistent cells inside of host tissues that survive antibiotic treatment and can re-colonize the antibiotic-liberated gut lumen post treatment [9, 82, 129, 130]. As with *C. difficile*, the relapse of *S*. *Typhimurium* in a mouse model can be prevented by co-housing with mice that did not receive antibiotic treatment, further showing that microbiota transfer limits recurring infections [131]. Microbiome restoration strategies following antibiotic treatment have also been explored in other body sites. For example, restoration of a healthy vaginal microbiome via microbiota transplantation has been shown to achieve long-term remission in 80% of patients who suffer from recurrent bacterial vaginosis infections [132].

The mechanisms by which microbiota transfer limits recurring infections after antibiotic treatment are likely diverse and mirror the mechanisms by which colonization resistance prevents strains from colonizing in the first place. Indeed, community restructuring after drug perturbations has been shown to be determined by shifts in the available nutrients, suggesting the alleviation of nutrient blocking. Similarly, the antimicrobial peptides RegIIIb and RegIIIy improve defence against gut pathogens like *S*. *Typhimurium*, *E. faecalis* and *Listeria monocytogenes* [133–135], but are depleted in response to antibiotic therapy [134]. As another example, antibiotic treatment depletes butyrate-producing microbes, such as those in the class *Clostridia*, which causes oxygen to leak into the gut and allows the expansion of facultatively anaerobic gut pathogens [136]. However, although the native microbiome can partially recover after antibiotic treatment, it remains unclear how these microbes survive antibiotic exposure or how recovery dynamics impact nutrient competition in cases of recurrent infection.

In addition to the effects of restoring competition in the microbiome after antibiotic treatment, the microbiome can modulate the ability of antibiotics to kill a focal species (Fig. 4; reviewed in [126]). Indeed, microbes can modify drugs via transformation to inactive forms [137] or bioaccumulation [138]. In a broad survey of microbe-drug interactions, a recent study profiled the effect of 30 drugs, including both non-antibiotics and antibiotics, on a 32-species community. Specifically, the authors compared the sensitivity of microbes to the drugs in communities compared to when grown in isolation [6]. The effect of the drug on microbes when in communities was different than when grown in isolation in more than a quarter of cases, highlighting the widespread nature of microbiome–drug interactions and underlining the importance of drug testing in the presence of communities. In that study [6], the most common outcome was cross-protection (Fig. 4a). A classic example is diffusible beta-lactamases that inactivate beta-lactam antibiotics and can lead to cross-protection of sensitive bacteria by resistant ones, as shown for *E. coli* protecting the pathogen *S*. *Typhimurium* from otherwise lethal concentrations of ampicillin [139]. Many gut microbiome species, such as *B. fragilis*, carry beta-lactamases [140], and, therefore, microbiome composition is expected to modulate the effectiveness of some antibiotics but not all. These effects will vary by the mechanism of resistance to a given antibiotic: for example, beta-lactamases act outside of the cell, meaning that resistance determinants can diffuse and cross-protect other microbes. In contrast, antibiotics such as fluoroquinolones or macrolides act on intracellular targets, meaning that resistance determinants are unlikely to diffuse and cross-protect other microbes. Microbe-microbe interactions can also influence the susceptibility of a pathogen to an antibiotic. For instance, sensitivity of *S*. *Typhimurium* to antibiotics can be reduced as a result of indole produced by *E. coli*, which causes oxidative stress and leads to increased antibiotic tolerance in *S*. *Typhimurium* [141].

**Fig. 4.**
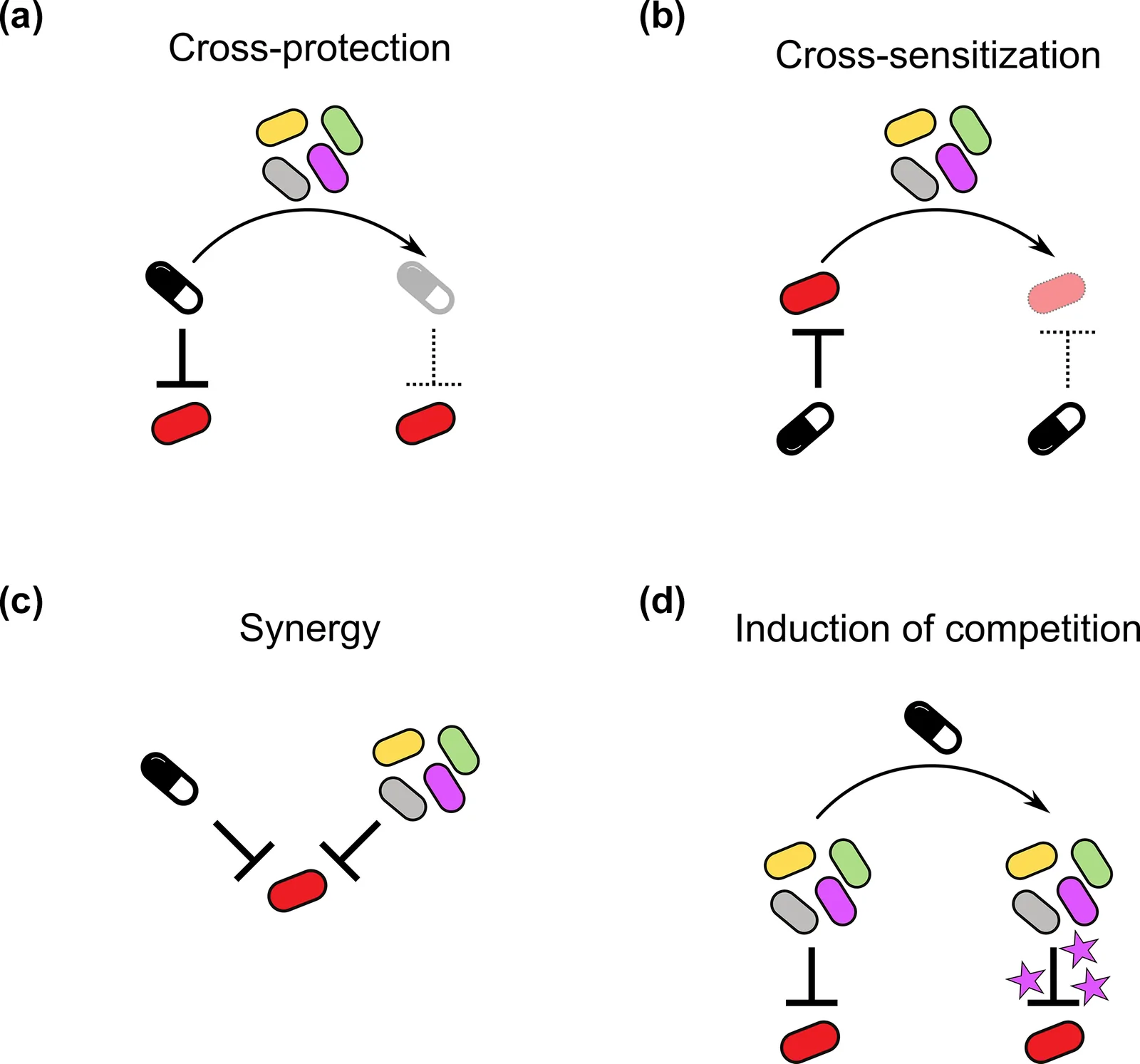
Microbiome ecology can modify the effectiveness of antibiotics. (**a**) Cross-protection. Microbes (multi-coloured rods) can reduce antibiotic (capsule) effectiveness by protecting susceptible pathogens (red rods), for example through antibiotic degradation, allowing pathogen survival despite treatment. (**b**) Cross-sensitization. In contrast, microbes can increase pathogen susceptibility to antibiotics, thereby enhancing antibiotic-mediated killing of the invader (faded red rod). (**c**) Synergy. Antibiotics and microbial competition can act together to suppress invading strains, resulting in enhanced clearance compared to either mechanism alone. (**d**) Induction of competition. Antibiotic treatment can promote the expression or activity of competition systems in microbes that can suppress pathogens, for example via interference competition (purple stars).

While cross-protection, as in the example of a beta-lactamase above, was more common, cross-sensitization also occurred [6] (Fig. 4b). Cross-sensitization of microbes to antibiotics can be via changes in cell regulation or be derived from nutrient dependencies. For example, if a microbe that cross-feeds another is killed by an antibiotic, it can collaterally lead to death of the other as the source of its metabolic dependency is gone, as demonstrated in synthetic communities [142]. The extent to which this occurs in natural microbiomes is yet to be determined, but cross-feeding networks are common in the gut microbiome [143]. Furthermore, as antibiotics can induce stress responses [144], they can trigger the expression of some mechanisms of interference competition like bacteriocins [145, 146], promoting the synergy between microbial competition and antibiotics (Fig. 4c, d).

Both cross-sensitization and cross-protection cases are interesting therapeutically. Cross-protection may improve the diversity of the microbiome and minimize collateral damage during treatment, while cross-sensitization may act to improve our ability to remove pathogens. While multiple examples of microbe–microbe and microbe–drug interactions affecting the effectiveness of antibiotics exist, future work is needed to better understand the mechanisms and contexts by which microbes modulate the ability of antibiotics to kill.

## Antibiotic-independent treatments

While antibiotics remain potent when resistance is absent, their collateral impacts on microbiomes have made antibiotic-independent alternatives tempting (Fig. 3c). And these strategies have the potential to turn the tide on infections even when resistance is rampant. One strategy is the administration of phages [147]. They represent self-replicating entities and can be considered analogous to mechanisms of bacterial warfare, as they can lead to cell lysis and modulate microbiomes [42, 147]. Consequently, administration of phages to microbiomes (phage therapy) can tip the balance between health and disease. For example, the administration of a phage cocktail was able to promote the decolonization of *K. pneumoniae* that promoted intestinal inflammation [148]. And, like many mechanisms of interference competition, phages are predominantly narrow in spectrum as they require specific cell-surface receptors for entry.

The adaptive immune response also has the potential to direct microbiome composition via the production of antibodies. In the intestine, IgA antibodies bind specific microbes [149], which can be raised towards a microbe of interest via vaccination and reduce its fitness in the gut [150]. However, this vaccine-derived IgA requires microbial competition to act in concert to facilitate decolonization of a target species. A recent study showed that vaccine-mediated decolonization of *E. coli* required both a specific IgA response as well as a metabolic niche competitor to provide nutrient competition [112]. But, not all microbes are easy to raise vaccines against, for example, those with thick capsule layers. A second recent study added that phages specific to the capsule of *E. coli* K1 can be used to promote a selective pressure for loss of the capsule, allowing vaccination to successfully raise antibodies against *E. coli* K1, which can then be eliminated using a niche competitor in parallel. On the skin, an antibody response generated by *S. epidermidis* could be redirected to target desired epitopes, such as the tetanus toxin [151]. Future work should address if this skin-directed vaccine can be used to engineer skin microbiomes.

Other strategies leverage small molecules, such as antisense oligonucleotides that can be delivered into microbes and interfere with specific mRNA sequences to inhibit translation of essential genes and kill target species [152]. In this case, targeting comes from sequence recognition, potentially allowing engineering in microbiomes while leaving the overall community intact, much like mechanisms of interference competition. As another approach, specificity can come from nanobodies. For example, chromosome-free bacterial cells have been engineered to encode nanobodies that can home these inert cells to a target and kill using T6SSs and reactive oxygen species [153]. In both these cases, the goal is to preserve the microbiome, which allows synergy with microbial competition to finish off a target strain that has been antagonized using these interventions, although future work is needed to evaluate the importance of microbial competition in these cases.

## Conclusions and outlook

We have argued that microbiome ecology is a critical determining factor for the invasion of AMR strains, the spread of AMR and the design of treatments to manage bacterial infections with or without antibiotics. We have discussed how there are parallels in the mechanisms that define the ability of a pathogen or AMR strain to colonize, the spread of AMR and reversal of pathogenic colonization. In all cases, mechanisms of microbial competition, such as resource and interference competition, are central. Importantly, these mechanisms do not act alone and instead synergize with each other and with other interventions, such as the administration of antibiotics, small inhibitory molecules or vaccines (Fig. 3) [34, 112, 131].

Our vision is that if we can identify the rank importance of different forms of microbial competition, their interaction and how microbiome context influences their effects, we can rationally engineer microbiomes to limit or reverse AMR. Such an approach could rely on a tailored combination of defined microbial consortia to maximize resource competition [13, 110, 111], targeted nutritional interventions [34, 154], engineered bacterial strains to encode bacterial weapons [34] and narrow-spectrum antimicrobials [11], phages or antibodies [112] to specifically target AMR strains. This would have the effect of minimizing AMR strain invasion and AMR spread or even facilitating decolonization, while preserving microbial diversity and, in turn, microbial competition. Here, more work is needed both experimentally to map how different strategies synergize and in which contexts, but also how we can predict how a particular combination of strategies may affect AMR spread. Mathematical models have the potential to make advances here, as they have the potential to bridge molecular, ecological and epidemiological scales to map the effects of microbiome interventions on AMR [155].

There are also still large gaps in our understanding of both what drives a successful strain in microbiomes, and how we can best apply interventions to embrace the competitive interactions within microbiomes. Additionally, while we now appreciate that perturbations to microbiomes can shift the probability of acquiring new strains that can boost the spread of AMR, we still are limited in our understanding of how we can predict microbiome restructuring after perturbations. Theory predicts that the type and strength of interaction between microbes influences stability in microbiomes [4], but we are far from being able to measure the distribution of all interactions, and even if we could measure all of them, they would be highly sensitive to context, as interactions can change in the presence or absence of other species.

Using the approach of seeking commonalities that drive success in microbiomes has potential to overcome these problems. For example, the necessity of microbial competition for nutrients and space has the potential to allow us to understand why a given species is successful in a niche or not. But, if we are to predict how competition for resources influences invasion of a strain and the spread of an AMR plasmid, we need to be able to map the available resources, such as nutrients, in a microbiome to the metabolism of each strain within it. Here, a recent study reported the use of mRNA translation prioritization as a proxy to determine microbial niches and predict strain invasion [156]. Our needs then also need to include understanding how perturbations, such as diet shifts in the gut, influence the available nutrients [15]. This is critical if we are to engineer microbiomes to be more protective against pathogens and the spread of AMR. In line with this, a recent study showed that the diet given to mice after antibiotic treatment is critical for the recovery of a microbiome [157].

An alternative approach to mapping nutrient utilization hierarchies and the available nutrients is to seek or design metabolic pathways and nutrient pairs that are unique for a given therapeutic microbe in a microbiome. This approach was used to successfully and reversibly engraft *B. thetaiotaomicron* in the mammalian gut. Specifically, porphyran, a marine polysaccharide, was supplemented to the diet and the locus for its utilization was engineered into *B. thetaiotaomicron* [154]. If we can successfully introduce microbes, we can equip them with mechanisms of interference competition to target AMR strains [34], or payloads that can cleave AMR genes. Here, a recent paper used a combination of a broad-spectrum conjugative plasmid and CRISPR-guided targeting of transposons to transfer large fragments of DNA to edit microbiomes *in situ* specifically [158], including potentially inactivating resistance genes.

Altogether, the application of ecology to microbiomes has the potential to provide tools to disentangle its complexity, guide strategies to engineer microbiomes and explain why some interventions succeed while others fail. Although the development of microbial therapeutics is met with optimism, we urge the importance of ecological context when designing and implementing strategies to achieve more precise, realistic and effective clinical outcomes.

## Abbreviations

AMR: antimicrobial resistance
ESBL: extended-spectrum beta lactamase
NDM-1: New Delhi metallo-β-lactamase-1
SCFA: short-chain fatty acid
T6SS: Type 6 Secretion Systems
