# Erratum: Microbiome ecology and antibiotic treatment failure: resistance spread, microbial competition and community engineering

**DOI:** 10.1099/mgen.0.001904

**Published:** 2026-11-11

**Authors:** Myles Wheeler, Erik Bakkeren

**Affiliations:** 1 Department of Biological Sciences, University of Calgary, Calgary, Alberta, Canada; 2 Calvin, Phoebe and Joan Snyder Institute for Chronic Diseases, Cumming School of Medicine, University of Calgary, Calgary, Alberta, Canada

In the originally published version of this article, two errors were identified in Fig. 3. An extraneous “(d)” label was inadvertently introduced into the nutrient competition inset. Figure 3 contains only three panels, labelled (a)–(c), as stated in the figure caption.

Additionally, in panel (c), an unintended community of microbes was inadvertently introduced next to “Antibiotics”

The corrected version of Fig. 3 is provided below.

**Figure:**
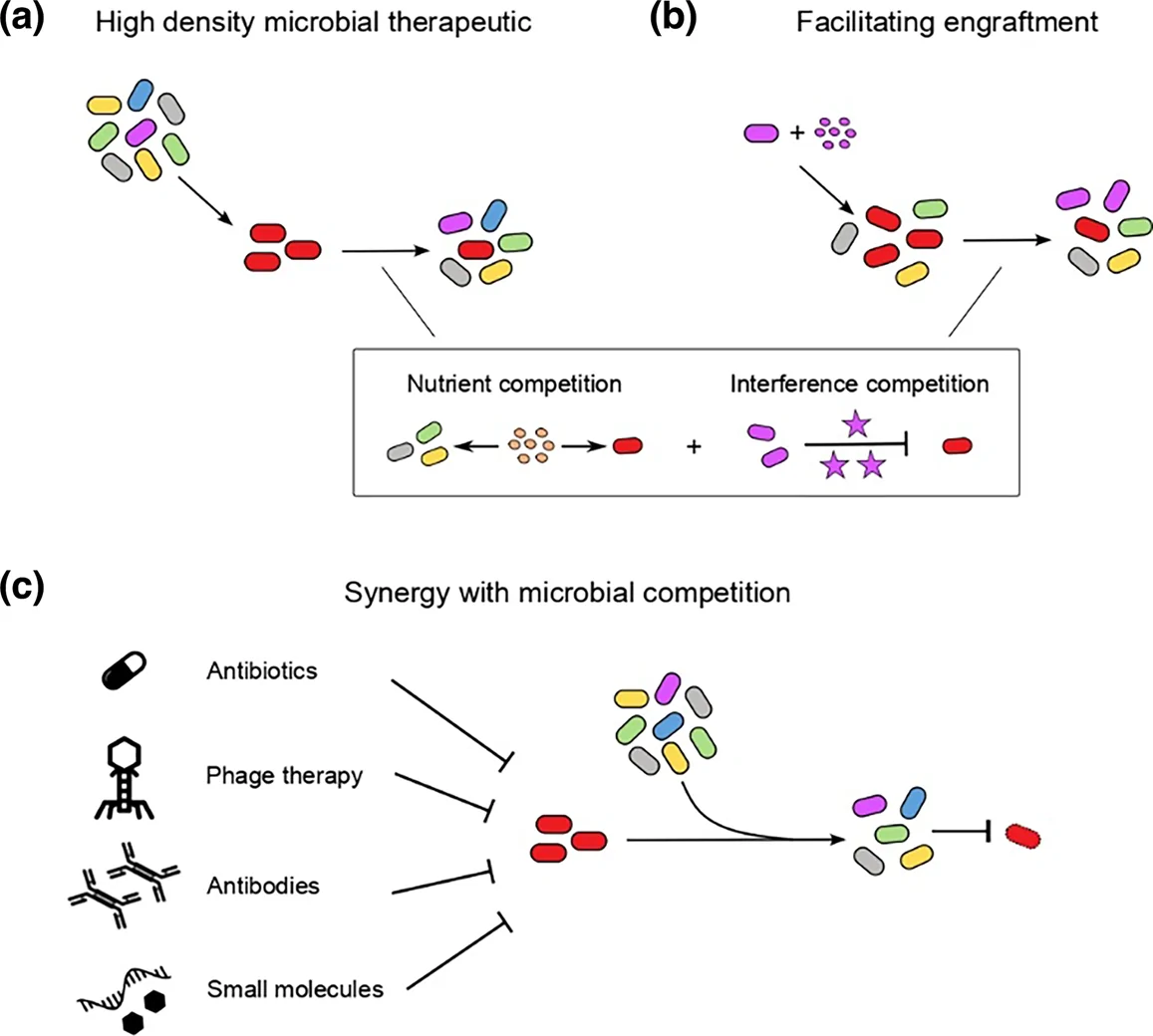


A further error was identified in the final paragraph of the section ‘Competition for resources is central to determining microbial invasion’, where the reference to ‘Fig. 1b” should read ‘Fig. 1a”

The Microbiology Society and the authors apologise for these errors.

